# A GATA3-specific DNAzyme attenuates sputum eosinophilia in eosinophilic COPD patients: a feasibility randomized clinical trial

**DOI:** 10.1186/s12931-018-0751-x

**Published:** 2018-04-04

**Authors:** Timm Greulich, Jens M. Hohlfeld, Petra Neuser, Katrin Lueer, Andreas Klemmer, Carmen Schade-Brittinger, Susanne Harnisch, Holger Garn, Harald Renz, Ursula Homburg, Jonas Renz, Anne Kirsten, Frauke Pedersen, Meike Müller, Claus F. Vogelmeier, Henrik Watz

**Affiliations:** 1 Department of Medicine, Pulmonary and Critical Care Medicine, University Medical Centre Giessen and Marburg, Philipps-University, Member of the German Centre for Lung Research (DZL), Marburg, Germany; 2 Department of Respiratory Medicine, Hannover Medical School, Fraunhofer Institute for Toxicology and Experimental Medicine, Member of the German Centre for Lung Research (DZL), Hannover, Germany; 3 Coordinating Center for Clinical Trials, Member of the German Centre for Lung Research (DZL), Philipps-University, Marburg, Germany; 4 Department of Medicine, Institute of Laboratory Medicine and Pathobiochemistry – Molecular Diagnostics, Member of the German Centre for Lung Research (DZL), Philipps-University, Marburg, Germany; 5 Sterna Biologicals GmbH & Co. KG, Marburg, Germany; 6 Pulmonary Research Institute at LungenClinic Grosshansdorf, Airway Research Center North (ARCN), Member of the German Centre for Lung Research (DZL), Grosshansdorf, Germany

**Keywords:** COPD, Sputum, Eosinophils, T-helper-2-cells, DNAzyme, SB010

## Abstract

**Background:**

A subset of COPD-patients presents with eosinophilic airway inflammation. While treatment of asthmatic patients with the GATA3-specific DNAzyme SB010 attenuated sputum eosinophilia after allergen challenge, this specific treatment has not been evaluated in patients with COPD. Our objective was to evaluate the feasibility and safety of inhaled SB010 in COPD patients with sputum eosinophilia.

**Methods:**

We conducted a randomized, double-blind, placebo-controlled, multicentre clinical trial in COPD-patients with sputum eosinophilia (≥2.5% non-squamous cells). Patients inhaled 10 mg SB010 bid or matching placebo via the controlled inhalation system AKITA2 APIXNEB for 28 days. Endpoints included the feasibility of the study (primary), patient’s safety, sputum eosinophils, F_E_NO, lung function, symptoms, and biomarkers. The study was registered in the German Clinical Trials Register: DRKS00006087.

**Results:**

One hundred thirty patients were screened, 23 patients were randomized (FEV_1_ 49.4 ± 11.5%; sputum eosinophils 8.0 ± 8.4%) and 19 patients completed the study (10 placebo, 9 SB010. After 28 days, SB010 decreased the relative sputum eosinophil count (*p* = 0.004) as compared to no changes in placebo-treated patients. F_E_NO, lung function, and symptoms were not affected significantly. We found an increase in blood IFN-γ (*p* = 0.02) and a trend to lower IL-5 levels in patients treated with SB010. SB010 was safe and well tolerated. Thirty five AEs (22 SB010, 13 placebo including 1 SAE) were observed with 3 AEs in each group judged to be possibly treatment-related.

**Conclusion:**

In patients with eosinophilic COPD, sputum eosinophils could be reduced by inhalation of SB010. Long-term studies are needed to demonstrate clinical efficacy.

**Electronic supplementary material:**

The online version of this article (10.1186/s12931-018-0751-x) contains supplementary material, which is available to authorized users.

## Background

Chronic obstructive pulmonary disease is characterized by persistent respiratory symptoms and an airflow limitation that is due to airway and/or alveolar abnormalities [1]. The airway inflammation of COPD patients is usually neutrophilic [2], but in a subgroup of COPD patients (up to 40%, depending on the definition) eosinophilic airway inflammation can be detected [3–5]. Sputum eosinophil count has been suggested as a biomarker for steroid responsiveness (inhaled and systemic) in these patients [6, 7].

Because eosinophil-mediated inflammation may play a role in COPD, it could be clinically useful to modify the biological pathways that evoke this type of inflammation. The zinc finger transcription factor GATA3 activates type 2 helper T cells (Th2 cells), leading to an increased production of interleukins- (IL) 4, 5, and 13 [8, 9]. IL-5, in turn, is known as the main driver for eosinophilic recruitment and activation [10, 11]. The same transcription factor GATA3 also acts in type 2 innate lymphoid cells (ILC2 cells) and leads to a production of a similar, even though not identical cytokine pattern, including IL-5, IL-13 and, to a lesser extent, IL-4 [12–14], which might mediate airway eosinophilia in nonallergic asthma and COPD [15]. In a recently published study, the anti-IL-5 monoclonal antibody mepolizumab led to a significant reduction of exacerbations in COPD patients with an elevated baseline blood eosinophil count [16].

DNA enzymes (DNAzymes) are single-stranded synthetic DNA antisense molecules that catalyse cleavage of specific RNA strands [17, 18]. The DNAzyme hgd40 (the active drug product in SB010) specifically binds to the messenger RNA (mRNA) of GATA3 and cleaves this target mRNA [19]. In murine models of allergic airway inflammation SB010 statistically significantly reduced GATA3 mRNA and subsequently led to a reduced production of Th2-specific cytokines [20]. In a recently published phase 2a trial, inhaled SB010 statistically significantly attenuated asthmatic responses in patients with allergic asthma and decreased Th2-mediated inflammatory profile including sputum eosinophilia [21, 22].

Here we assessed the feasibility of inhaling SB010 in patients with eosinophilic COPD for a larger subsequent trial. Furthermore, we evaluated the safety and efficacy of SB010 in this COPD subpopulation.

## Methods

### Study design and study population

We performed a randomized, double-blind, placebo-controlled, multicentre, phase 2a clinical study of SB010 in COPD patients with moderate to severe airflow obstruction (defined as a post-bronchodilator FEV_1_ of > 30%predicted to < 80%predicted and a post-bronchodilator FEV1/FVC less than 0.7) and the presence of sputum eosinophilia (≥2.5% non-squamous cells). The study was approved for all centres by the ethics committee at the University of Marburg as leading ethics committee (AZ: 149/13 A-ff, Jan 2014). After obtaining informed consent, patients were examined at a screening visit to assess eligibility for the trial (medical history, blood tests, drug testing, spirometry, sputum induction, and training for the AKITA inhalation device). Eligible patients were randomized in a 1:1 ratio by a central organisation (Centre for Clinical Trials, University of Marburg). During the following treatment period (28 days), patients inhaled 10 mg SB010 bid or matching placebo via the controlled inhalation system AKITA2 APIXNEB for 28 days. After the first inhalation of the investigational medicinal product (IMP, SB010) or placebo at day one, patients were closely monitored for 12 h at the centre, including regular assessment of lung function. Short clinical visits on a weekly basis (adverse events, vital signs, pharmacokinetic testing) ensured patient’s safety during the treatment period. At day 28, the last inhalation of the IMP was performed at the study centre. Subsequently, patients underwent serial lung function testing until 12 h after the inhalation manoeuvre. Induced sputum was performed at screening and day 29. A final follow-up visit was scheduled for 4 weeks later. Additional details regarding the study flow and the schedule of assessments can be found in Additional file 1: Figure S1 and Additional file 2: Table S1.

The primary endpoint was the feasibility of the study. Based on available literature regarding sputum eosinophilia in COPD (defined as sputum eosinophils ≥2.5%) and conservative estimates, we expected a screening to randomization ratio of 1:5 [23]. Exploratory endpoints included patient’s safety, sputum eosinophil count, fraction of exhaled nitric oxide (F_E_NO) at a flow rate of 50 ml/s, spirometry (FEV_1_ and FVC), symptom scores, and exploratory biomarkers.

Throughout the study, patients continued to use their concomitant COPD medication that could include inhaled corticosteroids. To exclude carry-over effects of changes in baseline medication, the concomitant medication had to be stable for at least one month before study start. Patients had to be able to inhale in an appropriate manner from the AKITA2 APIXNEB device. The main exclusion criteria were the presence of other relevant pulmonary diseases (e.g. history of asthma, known active tuberculosis, history of bronchiectasis) or history of thoracic surgery, a clinically relevant acute infection in the last 4 weeks prior to informed consent, chronic infections, and other. The complete list of inclusion and exclusion criteria is given in Additional file 3: Table S2.

### Assessments

The following assessments were performed according to current guidelines: spirometry (FEV_1_, FVC) [24], sputum induction [25], measurements of F_E_NO at a flow of 50 ml/s [26], pharmacokinetic measurements, COPD Assessment Test (CAT) [27], and St. George’s Respiratory Questionnaire (SGRQ) [28]. Exploratory biomarker analysis was performed in sputum and plasma (including signature cytokines for T cell subpopulations and a broad panel of pro-inflammatory innate cytokines and chemokines) as described in the supplemental methods. Time points for these assessments can be seen in Additional file 2: Table S1.

### Statistical analysis

Since the study represented a feasibility study, a formal sample size calculation was not conducted. A total of twenty included subjects were defined as sufficient to assess whether the study design would be suitable for a larger phase III clinical trial. Data are presented as mean ± standard deviation (SD) or percentage values unless stated otherwise. To assess within and between group differences in continuous variables, *P* values were calculated using the Wilcoxon signed rank test and exact Wilcoxon two-sample test, respectively. Because of the small sample size, *P* values were also calculated by the parametric equivalents (paired and independent *t* test) for verification but not reported. Differences between groups in categorical variables were tested using Fisher’s exact test. Tests were two-sided, *P* values < 0.05 were considered statistically significant. The software package SAS version 9.4 (SAS Institute Inc., Cary, NC, USA) was used for all statistical analyses, GraphPad Prism Version 7.01 (GraphPad Software, Inc., La Jolly, CA, USA) was used to draw the figures.

## Results

### Study population

**Fig. 1:**
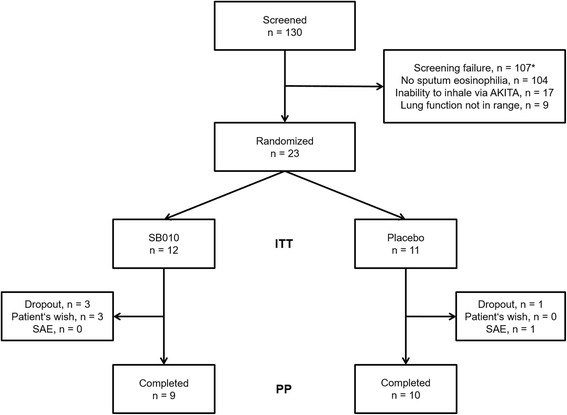
Trial Flow: 23 out of 130 screened patients (screening failure rate 82%) were randomized in a 1:1 ratio to receive SB010 (10 mg bid) or matching placebo. The dropout rate was 3/12 in the SB010 arm and 1/11 in the placebo arm. Reasons for dropout in the SB010 arm were not related to the study drug in 2 cases and may have been study drug-related in one case (mild AE in conjunction with patient’s wish). ITT: Intention-to-treat population; PP: Per-Protocol population. *Numbers for single screening failure reasons do not add up to total number, because two or more reasons could co-exist in one patient

Between August 2014 and August 2016, 130 patients were screened and 23 patients were enrolled (Fig. 1). The three most important reasons for screening failure (multiple reasons per patient possible) were missing sputum eosinophilia (*n* = 104), inability to inhale via the AKITA2 APIXNEB inhalation system (*n* = 17) and a post-bronchodilator lung function not meeting the inclusion criteria (FEV_1_/FVC <  0.7; FEV_1_ 30–80% predicted; *n* = 9). During the treatment period, 4 patients discontinued prematurely (Fig. 1).

Enrolled patients were predominantly male (15 male, 8 female), 63.3 ± 8.5 years of age, had a BMI of 25.8 ± 4.7, and a mean post-bronchodilator FEV_1_ 49.4% predicted. IgE levels were higher in the Placebo group, while patients treated with SB010 were slightly more eosinophilic (Table 1). Baseline characteristics of the intention-to-treat population (all enrolled patients, *n* = 23) are given in Table 1, while baseline characteristics of the per protocol population (patients that completed the 28 days of double-blind treatment, *n* = 19) are displayed in Additional file 4: Table S3.

**Table 1:** Baseline characteristics of the intention-to-treat population

	Placebo (*n* = 11)	SB010 (*n* = 12)	*p*-value
Gender [M/F]	7/4	8/4	n.s.
Age [years]	58.9 ± 6.9 59 (54–64)	67.4 ± 7.9 70 (65–73)	< 0.05
Height [cm]	172 ± 8 170 (167–176)	171 ± 13 172 (160–178)	n.s.
Weight [kg]	76.9 ± 21.6 73.5 (61–92.4)	75.5 ± 15.4 71.8 (66–82.5)	n.s.
BMI [kg/m^2^]	25.9 ± 6.4 25.5 (20.4–29.8)	25.6 ± 2.7 25.2 (23.4–28.0)	n.s.
FEV_1_ [l] post-bd	1.57 (1.17–1.78)	1.16 (0.98–1.72)	n.s.
FEV_1_ [% pred.] post-bd	51.5 ± 13 51 (40.1–60.9)	47.5 ± 10.2 46.4 (39–55.5)	n.s.
Reversibility (post-BD – pre-BD) [l]	0.17 (0.13–0.37)	0.13 (0.04–0.23)	n.s.#
GOLD stages [II/III]	6/5	4/8	n.s.
Current/Ex Smokers	8/3	7/5	n.s.
Packyears	46.1 ± 20.4 42.1 (30–70)	53.6 ± 29.9 45 (30.8–83.5)	n.s.
LAMA-containing regimen [%]	63.6	58.3	n.s.
LABA-containing regimen [%]	45.5	50.0	n.s.
ICS-containing treatment regimen [%]	54.6	41.7	n.s.
IgE_tot._ [U/ml]	75 (11–991.4)	22.6 (9.5–51.3)	n.s.
IgE_tot._ > 100 [%]	45.5	16.7	n.s.
FeNO [ppb]	22.0 (11.7–28.0)	13.7 (8.5–21.7)	n.s.
Blood eosinophils [G/l]	0.26 ± 0.11 0.24 (0.16–0.32)	0.28 ± 0.11 0.28 (0.21–0.36)	n.s.
Blood eosinophils [%]	3.5 ± 1.5 3.0 (2.2–4.9)	4.0 ± 1.6 4.1 (3.6–5.0)	n.s.
Sputum eosinophils [× 10^3^/ml]	86 (30–279)	245 (105–552)	n.s.
Sputum eosinophils [% non-squamous cells]	4.6 (3.3–10.1)	6.1 (4.2–7.9)	n.s.

Data are displayed as mean ± SD in the upper row, median (25% – 75%) in the lower row. For variables not normally distributed in at least one group, data are displayed as median (25% percentile – 75% percentile). Categorical variables are displayed as absolute numbers or percentage as indicated. *P* values were calculated using the exact Wilcoxon two-sample test for continuous variables and Fisher’s exact test for categorical variables As tests for normality of residuals were based on a small sample size, the *t* test was also performed for each continuous variable yielding the same result with regard to statistical significance except for reversibility. FeNO: Fraction of exhaled nitric oxide (at 50 ml/s flow-rate); *LABA* Long acting beta-2-receptor agonist, *LAMA* Long acting muscarinic acetylcholine-receptor antagonist, *ppb* Parts per billion, *ICS* Inhaled corticosteroid, *n.s* Not significant. # *p* < 0.05 tested with two-sample *t* test

### Exploratory efficacy analyses

**Fig. 2:**
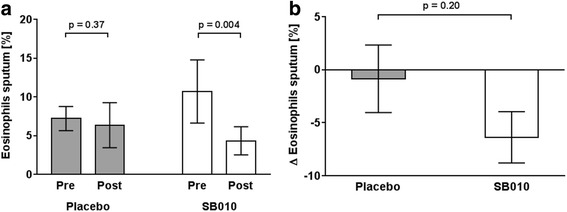
In the per-protocol population, SB010 led to a statistically significant reduction of the relative sputum eosinophils [% non-squamous cells], while placebo treatment did not change sputum eosinophil count (**a**). Comparing the deltas, no statistically significant difference was found (**b**). Data are displayed as mean ± standard error of the mean (SEM). *P* values were calculated by the two-sided Wilcoxon signed rank test for pre/post differences and the exact Wilcoxon two-sample test for the comparison of the deltas

Comparing sputum eosinophilia before and after 28 days of treatment in the per protocol population, SB010 significantly reduced the relative sputum eosinophil count (Fig. 2a, Table 2). By contrast, no significant changes occurred in placebo-treated patients (Fig. 2a, Table 2). Comparing the deltas (pre/post) between the two groups, we did not find a statistically significant difference (Fig. 2b). Similar results were obtained regarding the absolute number of sputum eosinophils, however, the effect was not statistically significant (SB010: *p* = 0.06; Placebo: *p* = 0.54; Table 2).

**Table 2:** Sputum cell counts (absolute and relative counts)

	Placebo (*n* = 10)			SB010 (*n* = 9)		
	Screening	d29	*p*-value	Screening	d29	*p*-value
Absolute cell counts [× 10^3^/ml]						
Total cell count	2854 ± 3259 2154 (935–3104)	3334 ± 2156 2691 (1712–4233)	0.27	6207 ± 7229 3509 (1782–7288)	6845 ± 5432 5628 (3684–6279)	1.00
Alevolar Macrophages	508 ± 555 312 (165–473)	509 ± 369 387 (263–721)	0.42	775 ± 711 539 (188–1040)	862 ± 808 611 (460–831)	0.65
Eosinophils	202 ± 225 88 (30–279)	174 ± 212 88 (22–206)	0.54	704 ± 1011 347 (107–544)	196 ± 134 192 (121–234)	0.06
Neutrophils	2083 ± 3160 1004 (464–2496)	2596 ± 1907 2048 (1166–3345)	0.08	4556 ± 6876 1828 (1429–4491)	5642 ± 5590 3366 (2828–5398)	0.65
Lymphocytes	20.3 ± 20.5 14 (8–29)	13.5 ± 17.6 9 (0–22)	0.84	29.6 ± 37.5 20 (8–29)	35 ± 45.7 16 (3–36)	0.73
Monocytes	4.6 ± 6.8 1 (0–10)	5.2 ± 4.5 4.5 (2–9)	0.73	6.8 ± 9.1 2 (0–9)	19.3 ± 37.9 0 (0–11)	0.80
Relative cell counts [% non-squamous cell]						
Alevolar Macrophages	23.8 ± 20.1 17.7 (8.4–41.7)	16.7 ± 9.4 14.2 (9.6–25.4)	0.49	19.8 ± 16.4 15.4 (11.2–21.9)	17.7 ± 14.6 14.8 (7.3–24.2)	0.57
Eosinophils	7.2 ± 4.9 5 (3.6–10.1)	6.4 ± 9.2 2 (1–7.1)	0.38	10.7 ± 12.3 6.5 (6–8)	4.4 ± 5.4 2.7 (1.4–4)	0.004
Neutrophils	65.2 ± 23.2 70.2 (49.6–82)	74.5 ± 18.1 82.1 (68.1–85.8)	0.13	65.2 ± 26.3 73.7 (66.6–80.2)	74.8 ± 17.9 80.6 (70.7–86)	0.10#
Lymphocytes	0.88 ± 0.78 0.75 (0.3–1.6)	0.4 ± 0.36 0.4 (0–0.5)	0.17	0.68 ± 0.58 0.6 (0.1–1)	0.62 ± 0.68 0.6 (0.1–0.8)	1.0
Monocytes	0.23 ± 0.33 0.05 (0–0.3)	0.14 ± 0.15 0.1 (0.1–0.1)	0.87	0.22 ± 0.39 0.1 (0–0.1)	0.38 ± 0.78 0 (0–0.4)	0.90

Displayed are the mean ± SD, the median (25% - 75%) in the second row; *P* values were calculated by the two-sided Wilcoxon signed rank test. All values are given for the per protocol population. As tests for normality were based on small sample sizes, paired *t* tests were also performed for each variable yielding the same results with regard to statistical significance except for neutrophils (%) in the SB010 group. # *p* < 0.05 tested with paired *t* test

Other cell types remained mainly unaffected by 28 days’ treatment with SB010 or matching placebo (Table 2).

**Fig. 3:**
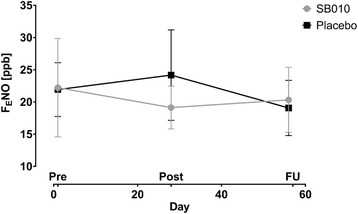
No statistically significant changes were observed in the FENO measurements during the trial. Data are displayed as mean ± SEM. Pre: Pre-Treatment; Post: Post-Treatment; FU: Follow-up

FENO levels decreased during the treatment period under SB010 treatment and slightly increased during follow-up, while an opposite trend was seen in placebo-treated patients (Fig. 3).

**Fig. 4:**
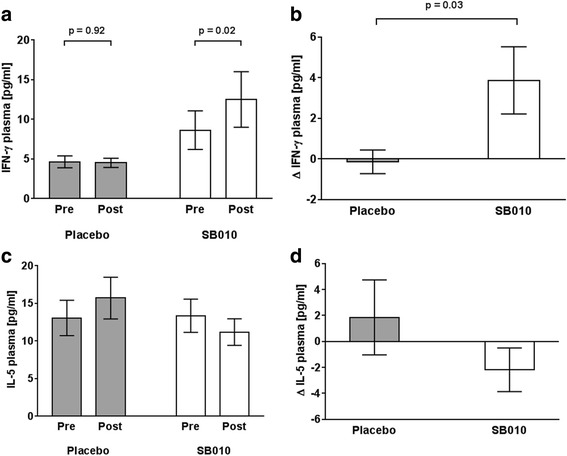
SB010 led to a statistically significant increase of plasma IFN-γ, that was not seen in placebo-treated patients (**a**). The deltas (pre/post) were statistically higher in SB010-treated patients as compared to placebo-treated patients (**b**). There was a trend towards a reduction of plasma IL-5 in the SB010 group that was opposite in the placebo group (**c**), however with no significant difference between the deltas (**d**). Data are displayed as mean ± SEM. P values were calculated by the two-sided Wilcoxon signed rank test for pre/post differences and the exact Wilcoxon two-sample test for the comparison of the deltas

To provide a mechanistic explanation for the SB010-associated reduction of sputum eosinophilia, we performed exploratory biomarker analysis in sputum and plasma before and after the treatment period (Additional file 2: Table S1). SB010 significantly increased plasma IFN-γ (*p* = 0.02; *p* = 0.03 for comparing the deltas), while this was not observed in placebo-treated patients (*p* = 0.92; Fig. 4a and b). A trend towards a reduction of plasma IL-5 levels was observed in the SB010 group that was opposite in the placebo group (4C); the difference between the deltas was not statistically significant (4D). IL-13 was not detectable in the majority of samples. Other exploratory biomarker analyses did not further clarify a potential mechanism of SB010 (Additional file 5: Table S4 and Additional file 6: Table S5).

Additionally, a broad spectrum of pro-inflammatory and chemotactic mediators was assessed in both blood and sputum, including pro-inflammatory cytokines IL-1α, IL-6, IL-18, and TNFα and the chemokines IL-8, MCP-1, MIP-1α and MIP-1β. Comparing day 1 with day 28, none of these mediators showed a statistically significant change under SB010 treatment. This may indicate that SB010 treatment did not induce and/or stimulate acute inflammatory responses related to the activation of innate immune cells (data not shown). Furthermore, no relevant differences were observed for sputum cytokine levels (Additional file 5: Table S4).

**Fig. 5:**
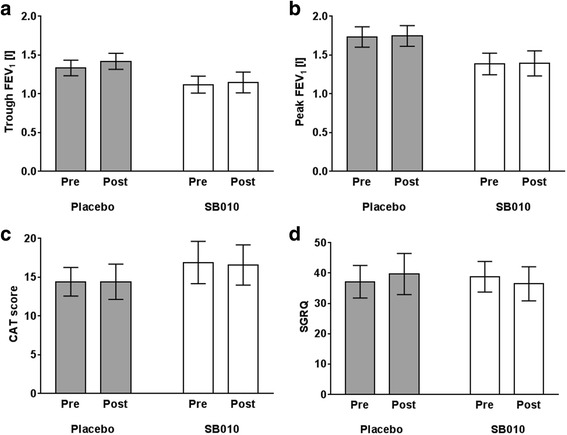
Neither SB010 nor placebo led to statistically significant differences in lung function (**a**, **b**), CAT (**c**), or SGRQ (**d**) before/after 28 days treatment. Data are displayed as mean ± SEM

Lung function (FEV_1_ trough or peak; Fig. 5a and b) or health status assessments (CAT or SGRQ; Fig. 5c and d) or blood eosinophils did not change statistically significantly in either group.

### Adverse events

Adverse events occurring at any time during the course of the study after the first administration of investigational drug were counted once per patient. If a patient experienced the same AE several times, only the highest grade was taken into account. Within the ITT population, 12 patients (five placebo, seven SB010) experienced at least one AE, three of these patients experienced one AE twice. A total of 35 AEs was reported with 22 AEs in SB010-treated patients and 13 in the placebo group. No obvious organ preference or clear pattern of repeated events was observed. In the organ class “respiratory, thoracic and mediastinal”, 5 events were observed in the SB010 group (4 grade 2) and 2 in the placebo group (2 grade 2). The number of adverse events that were rated as possibly, probably or certainly related to the study drug was identical (*n* = 3) in both groups. The only serious adverse event during the study (colitis that led to hospitalization) occurred in the placebo group. A detailed summary of adverse events can be found in Additional file 7: Table S6A and B, and Additional file 8: Table S7. In conclusion, SB010 was safe and well tolerated in the COPD treatment group.

## Discussion

To the best of our knowledge, this is the first study of a DNAzyme-based therapeutic approach in COPD patients. We could demonstrate that it is feasible to reduce sputum eosinophilia in the sputum eosinophil-high subgroups of COPD patients inhaling the GATA3-specific DNAzyme SB010 over a period of 4 weeks.

This was the first trial evaluating a GATA3-specific substance in a population of COPD patients with eosinophilic airway inflammation. We demonstrated the feasibility for a larger trial, as we met our expectations regarding the enrolment to screening ratio (we expected 1:5; we found a ratio of 1:5.7 with regard to eligibility and a ratio of 1:5 with regard to sputum eosinophils ≥2.5%). The relevance of an eosinophilic signal in COPD is a matter of debate, since sputum and blood eosinophils may serve as a biomarker for responsiveness towards inhaled corticosteroids [29–31] and anti-IL-5-directed therapies [16, 32]. Interestingly, it has been recognized that alternative pathways (Th2-mediated vs. ILC2-mediated pathways) can result in an eosinophilic airway inflammation [14, 15]. Because GATA3 is involved in both pathways, it seems reasonable to target this molecule when treating patients with an eosinophilic airway inflammation.

SB010 has been evaluated in asthmatic animal models and human studies: In a murine model of allergic airway inflammation, SB010 statistically significantly reduced GATA3 mRNA and led to a subsequently reduced production of Th2-specific cytokines [20]. In a recently published phase 2a trial, inhaled SB010 statistically significantly attenuated asthmatic responses after allergen challenge in patients with allergic asthma and decreased the Th2-mediated inflammatory profile including blood interleukin-5 and sputum eosinophilia [22]. This effect was more prominent in patients exhibiting higher baseline eosinophil blood count [21]. While both trials and disease entities are clearly different, it is interesting to see that the percentage point reduction of sputum eosinophilia based on relative sputum eosinophils (− 6.4%) observed in our trial is roughly of the same magnitude as seen in the asthma trial. To the best of our knowledge, this is the first time that an effect of a DNAzyme-based therapy has been observed in a COPD study population.

Comparing our treatment strategy to other anti-eosinophilic therapies in COPD, there is one study applying the interleukin-5 receptor antibody benralizumab available. Brightling et al. randomly assigned 101 patients to receive placebo or benralizumab. Benralizumab depleted the sputum eosinophil count, which was associated with a decrease of the number of exacerbations in the high eosinophilic subgroup of patients [32]. Another pair of clinical trials evaluated the interleukin-5 antibody mepolizumab in COPD. In a combined analysis, a greater effect of mepolizumab, as compared with placebo, on the annual rate of moderate or severe exacerbations was found among patients with higher blood eosinophil counts at screening [16]. In our study, SB010 markedly reduced the sputum eosinophil count (10.7 ± 12.3 to 4.4 ± 5.4; *p* = 0.004), but clinical efficacy (e.g. reduction of exacerbations) needs to be demonstrated in larger studies.

The marked reduction in sputum eosinophils was in part paralleled by a numerically slight decrease in F_E_NO, which increased again during follow-up; in placebo, an opposite trend was observed. In order to analyse the relationship between possible changes in adaptive T cell responses and the observed decrease in eosinophils following SB010 treatment, signature cytokines of Th1, Th2, Th17 and Treg cells were measured in patients’ plasma samples. A significant increase in IFN-γ (signature cytokine for Th1 T cells) levels were observed following SB010 treatment. In contrast, no significant changes were detected for the Th2 cytokine IL-5 (even though a trend for reduced IL-5 levels was observed), and IL-13 was not detectable in the majority of samples. Therefore, we have no clear indication whether SB010 treatment would change the qualitative or quantitative composition of Th2 cells in the peripheral blood of this patient population.

Regarding adverse events that were rated as possibly, probably or certainly related to the study drug, numbers were equal in both groups. The number of all reported adverse effects was higher in SB010 than in placebo. As no single event was experienced by more than two patients in either group (with the exception of headache, *n* = 3 in SB010), we do not regard this as an alarming signal. The slightly higher number of adverse events in the organ class “respiratory, thoracic and mediastinal” needs attention in further studies. Taken together, although slightly higher numbers of AEs occurred in the verum group as compared to placebo, SB010 seemed to be safe and was not associated with any serious adverse events in the studied COPD population.

Our study clearly has limitations. First, it is a small number of patients included. This resulted in marked differences in the baseline characteristics of both treatment arms, being the baseline sputum eosinophil count the most important one. Therefore, the observed effect of the active treatment could be seen as regression towards the mean rather than a clear direct effect on sputum eosinophilia. On the other hand, all individual relative sputum eosinophil counts decreased in SB010-treated patients while an undirected change was observed in placebo-treated patients (Additional file 9: Figure S2a-c). Further differences between both arms included a strikingly higher serum IgE count in the placebo arm which was associated with a higher reversibility (median 0.17 L after bronchodilation as compared to median 0.13 L in the SB010 arm). However, if these discrepancies influenced the results, we would expect that his would have decreased the “real” effect, as higher IgE and higher reversibility would reflect a higher variability and – maybe – a higher responsibility for Th2-targeted therapy. A further point of discussion is whether COPD patients with eosinophilic airway inflammation may be denominated more precisely as smoking asthma patients [1, 33, 34]. However, asthma patients (based on their clinical history) were excluded from the trial, and the percentage of patients being on inhaled corticosteroids was not higher than in other interventional or observational studies [35, 36]. The strength of our study is that we were able to confirm that - based on a biomarker signal of elevated sputum eosinophils - a reduction in airway eosinophilia can be achieved by the GATA3-specific DNAzyme similar to the reduction observed in in asthma patients before [21, 22]. This provides further evidence that the GATA3 pathway is also involved in patients with eosinophilic COPD.

In summary, we demonstrated the feasibility of a study of a DNAzyme in a subgroup of COPD. The results of this phase 2a, randomized, double-blind, placebo-controlled, multicentre clinical trial of SB010 in COPD patients with sputum eosinophilia strengthen the hypothesis that a Th2-regulated airway inflammation can be modified in a subgroup of COPD patients.

## Conclusions

The results of our randomized clinical trial in COPD patients with sputum eosinophilia demonstrate that a Th2-regulated airway inflammation can be effectively attenuated by inhalation of the GATA3-specific DNAzyme SB010. Further studies with a larger number of patients and longer duration of treatment are needed to further assess clinical efficacy (e.g. reduction of exacerbations) and long-term safety in patients with this phenotype of eosinophilic COPD.

## Additional files

Additional file 1: **Figure S1.** Study Design: After the informed consent and screening visits patients were randomized in a 1:1 ratio and treated with SB010 mg bid or matching placebo via AKITA inhalation for 28 days. A follow-up visit was conducted 4 weeks after termination of IMP treatment. (JPEG 48 kb)

Additional file 2: **Table S1.** Study flow chart. (a) See section Methods for details. (b) In women with childbearing potential. (c) For cell differential and exploratory markers (d) Washout periods: SABA: 4 hours, LABA: 12 hours, Ultra-LABA: 24 h, Theophylline: 24 hours SAMA: 4 hours, LAMA: 24 hours, spirometry was performed before and after 20 min (± 2 min) after 400 μl Salbutamol) (e) Kit for 1 week plus 4 vials as reserve (f) Patients may stay overnight at study site. (g) ECP, IL-8, IFN-γ, Il1-β, Il-2, Il-6, Il-10, Il12p70, Il-13, TNF-α, TGF-β1, TPS, IFN-α2a, Il-17a, Il-18, Il-1α, Il- 22, Il-5, MCP-1, MIP-1β (h) Inhaled concomitant medication other than IMP will be continued throughout the study. (TIFF 198 kb)

Additional file 3: **Table S2.** Complete list of inclusion/exclusion-criteria. (JPEG 67 kb)

Additional file 4: **Table S3 ** provides the baseline characteristics of the per-protocol population. Data are displayed as mean ± SD in the upper row, median (25% percentile – 75% percentile) in the lower row. Categorical variables are displayed as absolute numbers or percentage as indicated. (TIFF 185 kb)

Additional file 5: **Tables S4A** and **S4B** give an overview of adverse events (A) and adverse reactions (relation to investigational drug: certain, possible, or probable) (B) that occurred after first administration of IMP, counted once per patient (highest grade). 12 patients (five placebo, seven SB010) experienced at least one AE, three of these patients experienced one AE repeatedly (twice). Five patients (two placebo, three SB010) experienced at least one AR, one of these patients experienced two different ARs, another patient the same AR twice. (TIFF 218 kb)

Additional file 6: **Table S5.** Supplement table 5 provides a detailed overview of all adverse events that occurred after first administration of IMP, counted once per patient (highest grade). 12 patients (five placebo, seven SB010) experienced at least one AE, three of these patients experienced one AE repeatedly (twice). N (number of patients who experienced the specified AE and grade in each group), percentages refer to n (number of patients in each group); N_SOC (minimum number of AEs occurring in the specified system organ class in both groups; N_PT (number of patients in both groups who experienced the specified AE). (TIFF 209 kb)

Additional file 7: **Tables S6 ** provides results of exploratory biomarker measurements in sputum (unit: pg/ml). Displayed are the mean ± SD, the median (25% – 75%) in the second row; *P* values were calculated by the two-sided Wilcoxon signed rank test. (JPEG 30 kb)

Additional file 8: **Table S7** provides results of exploratory biomarker measurements in plasma (unit: pg/ml). Displayed are the mean ± SD, the median (25% – 75%) in the second row; *P* values were calculated by the two-sided Wilcoxon signed rank test. (JPEG 65 kb)

Additional file 9: **Figure S2.** Displayed are the individual data of the relative sputum eosinophil count before and after 28 days treatment with placebo or SB010. The corresponding deltas are displayed in B, demonstrating that all relative sputum counts decreased under SB010 treatment while this was not the case in placebo-treated patients. We performed an outlier-analysis removing the very high eosinophils patient from panel B, showing the reduction in relative sputum eosinophils still being significant (*p* = 0.008; Wilcoxon signed rank test). (TIFF 178 kb)

## Abbreviations

AE: Adverse events
BMI: Body mass index
CAT: COPD assessment test
COPD: Chronic obstructive pulmonary disease
DNAzyme: Deoxyribozyme
F_E_NO: Fractional exhaled nitric oxide
FEV_1_: Forced expiratory volume in one second
FVC: Forced vital capacity
GATA3: Guanine adenine thymine adenine sequence-binding protein 3
hgd40: Human GATA-3 DNAzyme 40
IFN-γ: Interferon gamma
IgE: Immunglobulin E
IL: Interleukin
ILC2 (cells): Type 2 innate lymphoid cells
IMP: Investigational medicinal product
ITT: Intention-to-treat population
MCP-1: Monocyte chemoattractant protein-1
MIP-1α and MIP-1β: Macrophage inflammatory protein-1alpha or 1beta
mRNA: Messenger ribonucleic acid
SAE: Serious adverse event
SB010: Drug formulation of hgd40 for inhaled application
SD: Mean ± standard deviation
SGRQ: St. George’s respiratory questionnaire
Th1/Th2 (cells): T helper type 1 or 2 (cells)
TNFα: Tumor necrosis factor alpha
Treg: T regulatory cells

## References

[1] Vogelmeier CF, Criner GJ, Martinez FJ, Anzueto A, Barnes PJ, Bourbeau J, Celli BR, Chen R, Decramer M, Fabbri LM, et al. Global strategy for the diagnosis, management, and prevention of chronic obstructive lung disease 2017 report: GOLD executive summary. Eur Respir J. 2017;49(3). 10.1183/13993003.00214-2017 28182564

[2] Hogg JC, Chu F, Utokaparch S, Woods R, Elliott WM, Buzatu L, Cherniack RM, Rogers RM, Sciurba FC, Coxson HO, Pare PD (2004). The nature of small-airway obstruction in chronic obstructive pulmonary disease. N Engl J Med.

[3] Bafadhel M, McCormick M, Saha S, McKenna S, Shelley M, Hargadon B, Mistry V, Reid C, Parker D, Dodson P (2012). Profiling of sputum inflammatory mediators in asthma and chronic obstructive pulmonary disease. Respiration.

[4] Bafadhel M, Saha S, Siva R, McCormick M, Monteiro W, Rugman P, Dodson P, Pavord ID, Newbold P, Brightling CE (2009). Sputum IL-5 concentration is associated with a sputum eosinophilia and attenuated by corticosteroid therapy in COPD. Respiration.

[5] Singh D, Kolsum U, Brightling CE, Locantore N, Agusti A, Tal-Singer R, investigators E (2014). Eosinophilic inflammation in COPD: prevalence and clinical characteristics. Eur Respir J.

[6] Brightling CE, McKenna S, Hargadon B, Birring S, Green R, Siva R, Berry M, Parker D, Monteiro W, Pavord ID, Bradding P (2005). Sputum eosinophilia and the short term response to inhaled mometasone in chronic obstructive pulmonary disease. Thorax.

[7] Siva R, Green RH, Brightling CE, Shelley M, Hargadon B, McKenna S, Monteiro W, Berry M, Parker D, Wardlaw AJ, Pavord ID (2007). Eosinophilic airway inflammation and exacerbations of COPD: a randomised controlled trial. Eur Respir J.

[8] Ray A, Cohn L (1999). Th2 cells and GATA-3 in asthma: new insights into the regulation of airway inflammation. J Clin Invest.

[9] Zhang DH, Yang L, Ray A (1998). Differential responsiveness of the IL-5 and IL-4 genes to transcription factor GATA-3. J Immunol.

[10] Foster PS, Hogan SP, Matthaei KI, Young IG (1997). Interleukin-4 and interleukin-5 as targets for the inhibition of eosinophilic inflammation and allergic airways hyperreactivity. Mem Inst Oswaldo Cruz.

[11] Sur S, Gleich GJ, Swanson MC, Bartemes KR, Broide DH (1995). Eosinophilic inflammation is associated with elevation of interleukin-5 in the airways of patients with spontaneous symptomatic asthma. J Allergy Clin Immunol.

[12] Walker JA, Barlow JL, AN MK (2013). Innate lymphoid cells--how did we miss them?. Nat Rev Immunol.

[13] Spits H, Artis D, Colonna M, Diefenbach A, Di Santo JP, Eberl G, Koyasu S, Locksley RM, McKenzie AN, Mebius RE (2013). Innate lymphoid cells--a proposal for uniform nomenclature. Nat Rev Immunol.

[14] Brusselle GG, Maes T, Bracke KR (2013). Eosinophils in the spotlight: eosinophilic airway inflammation in nonallergic asthma. Nat Med.

[15] Bel EH, Ten Brinke A (2017). New anti-eosinophil drugs for asthma and COPD: targeting the trait!. Chest..

[16] Pavord ID, Chanez P, Criner GJ, Kerstjens HAM, Korn S, Lugogo N, Martinot JB, Sagara H, Albers FC, Bradford ES (2017). Mepolizumab for eosinophilic chronic obstructive pulmonary disease. N Engl J Med.

[17] Santiago FS, Khachigian LM (2001). Nucleic acid based strategies as potential therapeutic tools: mechanistic considerations and implications to restenosis. J Mol Med (Berl).

[18] Cho EA, Moloney FJ, Cai H, Au-Yeung A, China C, Scolyer RA, Yosufi B, Raftery MJ, Deng JZ, Morton SW (2013). Safety and tolerability of an intratumorally injected DNAzyme, Dz13, in patients with nodular basal-cell carcinoma: a phase 1 first-in-human trial (DISCOVER). Lancet.

[19] Sel S, Wegmann M, Dicke T, Sel S, Henke W, Yildirim AO, Renz H, Garn H (2008). Effective prevention and therapy of experimental allergic asthma using a GATA-3-specific DNAzyme. J Allergy Clin Immunol.

[20] Turowska A, Librizzi D, Baumgartl N, Kuhlmann J, Dicke T, Merkel O, Homburg U, Hoffken H, Renz H, Garn H (2013). Biodistribution of the GATA-3-specific DNAzyme hgd40 after inhalative exposure in mice, rats and dogs. Toxicol Appl Pharmacol.

[21] Krug N, Hohlfeld JM, Buhl R, Renz J, Garn H, Renz H. Blood eosinophils predict therapeutic effects of a GATA3-specific DNAzyme in asthma patients. J Allergy Clin Immunol. 2017;140(2):625–628.e5. 10.1016/j.jaci.2017.02.024 28342914

[22] Krug N, Hohlfeld JM, Kirsten AM, Kornmann O, Beeh KM, Kappeler D, Korn S, Ignatenko S, Timmer W, Rogon C (2015). Allergen-induced asthmatic responses modified by a GATA3-specific DNAzyme. N Engl J Med.

[23] Saha S, Brightling CE (2006). Eosinophilic airway inflammation in COPD. Int J Chron Obstruct Pulmon Dis.

[24] Brusasco V, Crapo R, Viegi G (2007). Coming together: the ATS/ERS consensus on clinical pulmonary function testing. Rev Mal Respir.

[25] Pedersen F, Holz O, Lauer G, Quintini G, Kiwull-Schone H, Kirsten AM, Magnussen H, Rabe KF, Goldmann T, Watz H (2015). Multi-analyte profiling of inflammatory mediators in COPD sputum--the effects of processing. Cytokine.

[26] American Thoracic Society; European Respiratory Society. ATS/ERS recommendations for standardized procedures for the online and offline measurement of exhaled lower respiratory nitric oxide and nasal nitric oxide, 2005. Am J Respir Crit Care Med 2005, 171**:**912–930. 10.1164/rccm.200406-710ST 15817806

[27] Jones PW, Harding G, Berry P, Wiklund I, Chen WH, Kline LN (2009). Development and first validation of the COPD assessment test. Eur Respir J.

[28] Jones PW, Quirk FH, Baveystock CM (1991). The St George's respiratory questionnaire. Respir Med.

[29] Brightling CE, Monteiro W, Ward R, Parker D, Morgan MD, Wardlaw AJ, Pavord ID (2000). Sputum eosinophilia and short-term response to prednisolone in chronic obstructive pulmonary disease: a randomised controlled trial. Lancet.

[30] Barnes NC, Sharma R, Lettis S, Calverley PM (2016). Blood eosinophils as a marker of response to inhaled corticosteroids in COPD. Eur Respir J.

[31] Watz H, Tetzlaff K, Wouters EF, Kirsten A, Magnussen H, Rodriguez-Roisin R, Vogelmeier C, Fabbri LM, Chanez P, Dahl R (2016). Blood eosinophil count and exacerbations in severe chronic obstructive pulmonary disease after withdrawal of inhaled corticosteroids: a post-hoc analysis of the WISDOM trial. Lancet Respir Med.

[32] Brightling CE, Bleecker ER, Panettieri RA, Bafadhel M, She D, Ward CK, Xu X, Birrell C, van der Merwe R (2014). Benralizumab for chronic obstructive pulmonary disease and sputum eosinophilia: a randomised, double-blind, placebo-controlled, phase 2a study. Lancet Respir Med.

[33] Woodruff PG, Agusti A, Roche N, Singh D, Martinez FJ (2015). Current concepts in targeting chronic obstructive pulmonary disease pharmacotherapy: making progress towards personalised management. Lancet.

[34] Wurst KE, Kelly-Reif K, Bushnell GA, Pascoe S, Barnes N (2016). Understanding asthma-chronic obstructive pulmonary disease overlap syndrome. Respir Med.

[35] Worth H, Buhl R, Criee CP, Kardos P, Mailander C, Vogelmeier C (2016). The 'real-life' COPD patient in Germany: the DACCORD study. Respir Med.

[36] Buhl R, Maltais F, Abrahams R, Bjermer L, Derom E, Ferguson G, Flezar M, Hebert J, McGarvey L, Pizzichini E (2015). Tiotropium and olodaterol fixed-dose combination versus mono-components in COPD (GOLD 2-4). Eur Respir J.

