# Supplementary figures and images for: A GATA3-specific DNAzyme attenuates sputum eosinophilia in eosinophilic COPD patients: a feasibility randomized clinical trial
Source: Respir Res. 2018 Apr 4;19:55. doi: 10.1186/s12931-018-0751-x (PMC5883532; doi:10.1186/s12931-018-0751-x)

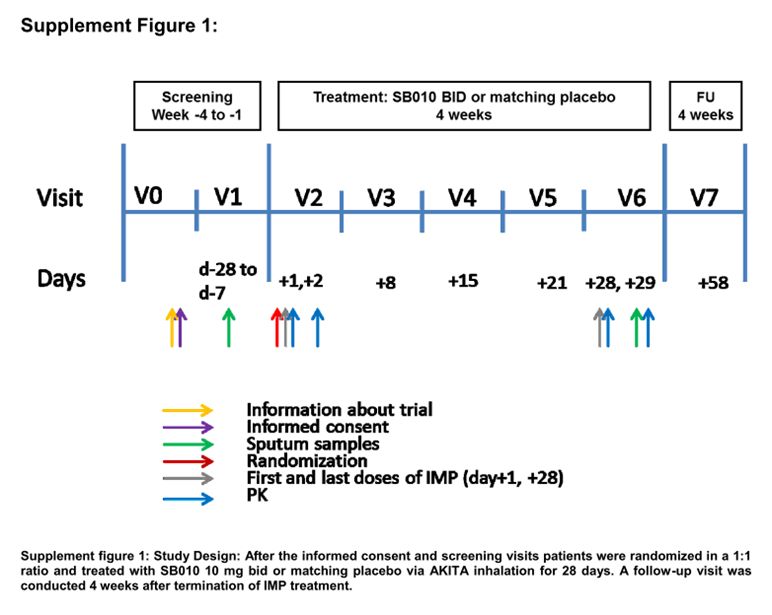

Supplement: Supplementary file 1 — Figure S1. Study Design: After the informed consent and screening visits patients were randomized in a 1:1 ratio and treated with SB010 mg bid or matching placebo via AKITA inhalation for 28 days. A follow-up visit was conducted 4 weeks after termination of IMP treatment. (JPEG 48 kb) [file 12931_2018_751_MOESM1_ESM.jpg]

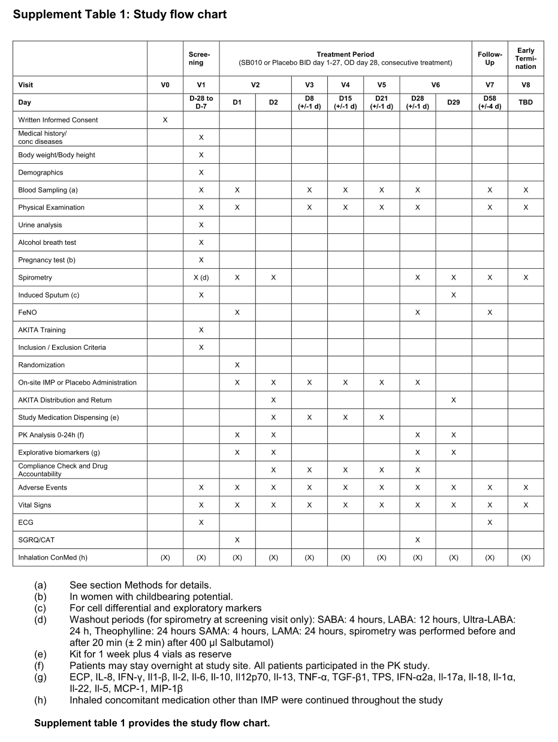

Supplement: Supplementary file 2 — Table S1. Study flow chart. (a) See section Methods for details. (b) In women with childbearing potential. (c) For cell differential and exploratory markers (d) Washout periods: SABA: 4 hours, LABA: 12 hours, Ultra-LABA: 24 h, Theophylline: 24 hours SAMA: 4 hours, LAMA: 24 hours, spirometry was performed before and after 20 min (± 2 min) after 400 μl Salbutamol) (e) Kit for 1 week plus 4 vials as reserve (f) Patients may stay overnight at study site. (g) ECP, IL-8, IFN-γ, Il1-β, Il-2, Il-6, Il-10, Il12p70, Il-13, TNF-α, TGF-β1, TPS, IFN-α2a, Il-17a, Il-18, Il-1α, Il- 22, Il-5, MCP-1, MIP-1β (h) Inhaled concomitant medication other than IMP will be continued throughout the study. (TIFF 198 kb) [file 12931_2018_751_MOESM2_ESM.tif]

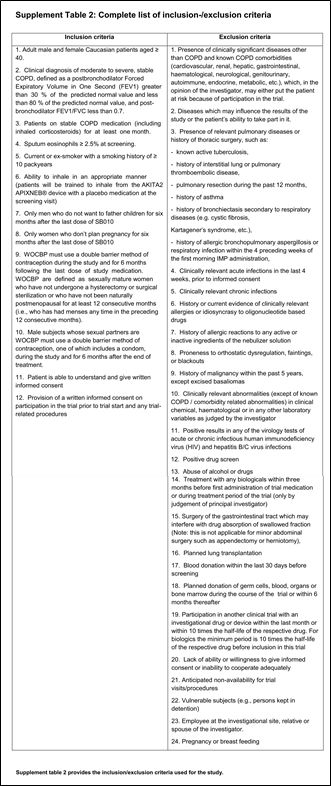

Supplement: Supplementary file 3 — Table S2. Complete list of inclusion/exclusion-criteria. (JPEG 67 kb) [file 12931_2018_751_MOESM3_ESM.jpg]

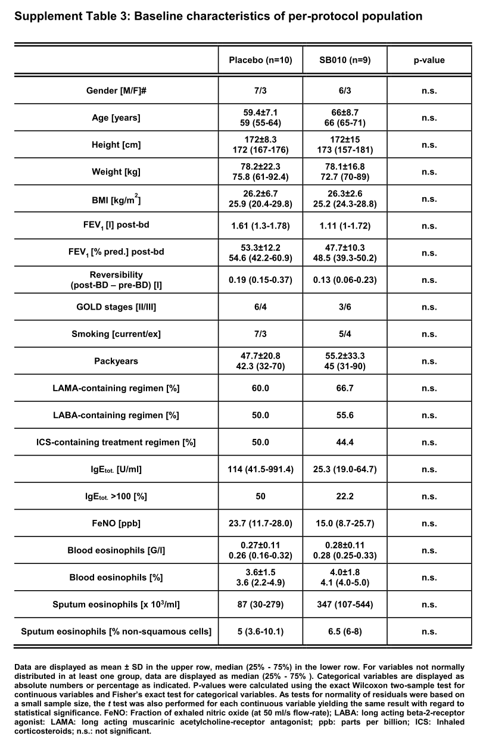

Supplement: Supplementary file 4 — Table S3 provides the baseline characteristics of the per-protocol population. Data are displayed as mean ± SD in the upper row, median (25% percentile – 75% percentile) in the lower row. Categorical variables are displayed as absolute numbers or percentage as indicated. (TIFF 185 kb) [file 12931_2018_751_MOESM4_ESM.tif]

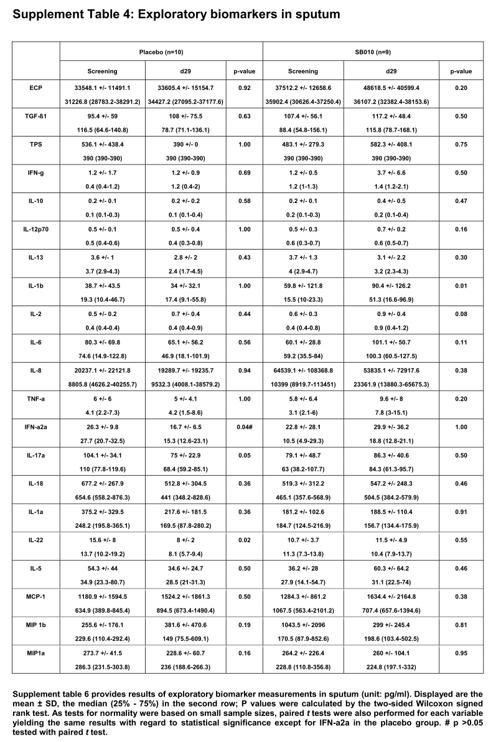

Supplement: Supplementary file 5 — Tables S4A and S4B give an overview of adverse events (A) and adverse reactions (relation to investigational drug: certain, possible, or probable) (B) that occurred after first administration of IMP, counted once per patient (highest grade). 12 patients (five placebo, seven SB010) experienced at least one AE, three of these patients experienced one AE repeatedly (twice). Five patients (two placebo, three SB010) experienced at least one AR, one of these patients experienced two different ARs, another patient the same AR twice. (TIFF 218 kb) [file 12931_2018_751_MOESM5_ESM.tif]

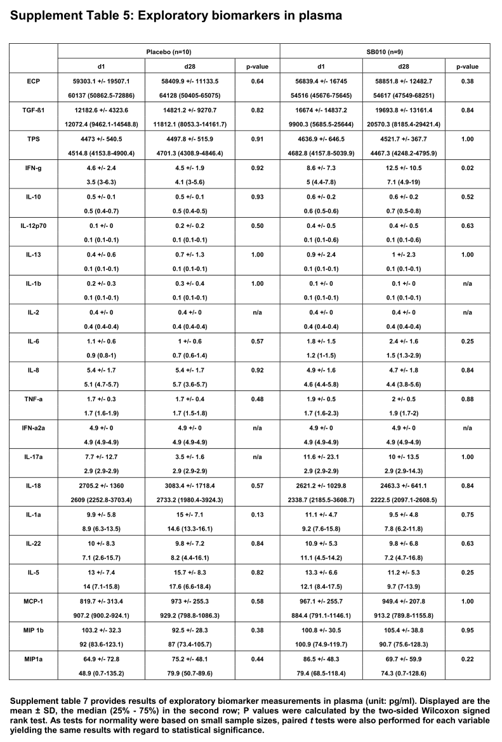

Supplement: Supplementary file 6 — Table S5. Supplement table 5 provides a detailed overview of all adverse events that occurred after first administration of IMP, counted once per patient (highest grade). 12 patients (five placebo, seven SB010) experienced at least one AE, three of these patients experienced one AE repeatedly (twice). N (number of patients who experienced the specified AE and grade in each group), percentages refer to n (number of patients in each group); N_SOC (minimum number of AEs occurring in the specified system organ class in both groups; N_PT (number of patients in both groups who experienced the specified AE). (TIFF 209 kb) [file 12931_2018_751_MOESM6_ESM.tif]

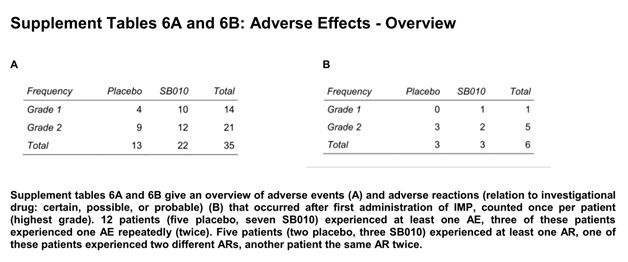

Supplement: Supplementary file 7 — Tables S6 provides results of exploratory biomarker measurements in sputum (unit: pg/ml). Displayed are the mean ± SD, the median (25% – 75%) in the second row; P values were calculated by the two-sided Wilcoxon signed rank test. (JPEG 30 kb) [file 12931_2018_751_MOESM7_ESM.jpg]

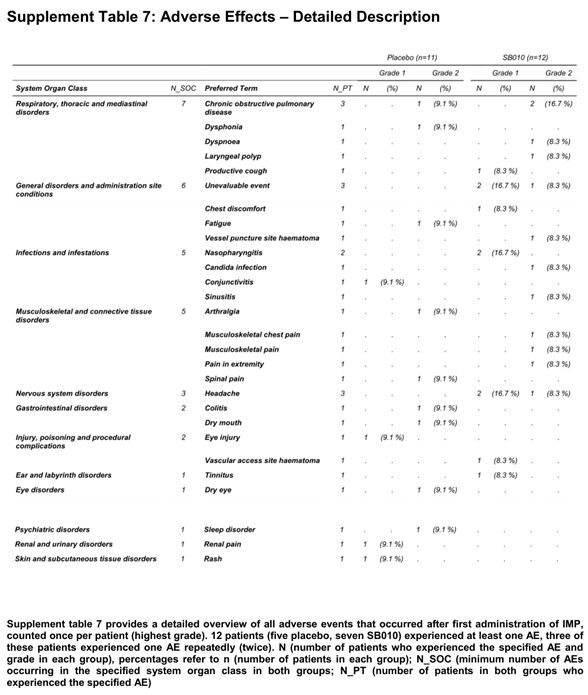

Supplement: Supplementary file 8 — Table S7 provides results of exploratory biomarker measurements in plasma (unit: pg/ml). Displayed are the mean ± SD, the median (25% – 75%) in the second row; P values were calculated by the two-sided Wilcoxon signed rank test. (JPEG 65 kb) [file 12931_2018_751_MOESM8_ESM.jpg]

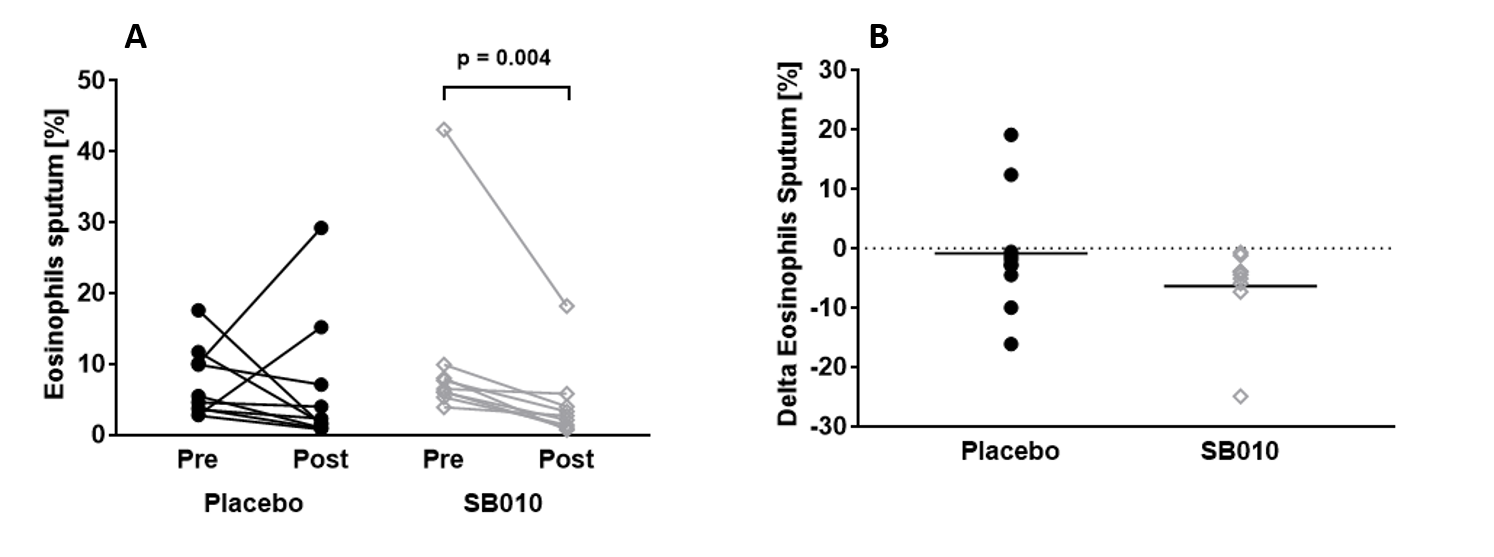

Supplement: Supplementary file 9 — Figure S2. Displayed are the individual data of the relative sputum eosinophil count before and after 28 days treatment with placebo or SB010. The corresponding deltas are displayed in B, demonstrating that all relative sputum counts decreased under SB010 treatment while this was not the case in placebo-treated patients. We performed an outlier-analysis removing the very high eosinophils patient from panel B, showing the reduction in relative sputum eosinophils still being significant (p = 0.008; Wilcoxon signed rank test). (TIFF 178 kb) [file 12931_2018_751_MOESM9_ESM.tif]
